# ChatGPT’s Response Consistency: A Study on Repeated Queries of Medical Examination Questions

**DOI:** 10.3390/ejihpe14030043

**Published:** 2024-03-08

**Authors:** Paul F. Funk, Cosima C. Hoch, Samuel Knoedler, Leonard Knoedler, Sebastian Cotofana, Giuseppe Sofo, Ali Bashiri Dezfouli, Barbara Wollenberg, Orlando Guntinas-Lichius, Michael Alfertshofer

**Affiliations:** 1 Department of Otorhinolaryngology, Head and Neck Surgery, University Hospital Jena, Friedrich Schiller University Jena, Am Klinikum 1, 07747 Jena, Germany; orlando.guntinas@med.uni-jena.de; 2 Department of Otolaryngology, Head and Neck Surgery, School of Medicine and Health, Technical University of Munich (TUM), Ismaningerstrasse 22, 81675 Munich, Germanyali.bashiri@tum.de (A.B.D.); barbara.wollenberg@tum.de (B.W.); 3 Department of Plastic Surgery and Hand Surgery, Klinikum Rechts der Isar, Technical University of Munich (TUM), Ismaningerstrasse 22, 81675 Munich, Germany; 4 Division of Plastic and Reconstructive Surgery, Massachusetts General Hospital, Harvard Medical School, 55 Fruit Street, Boston, MA 02114, USA; 5 Department of Dermatology, Erasmus Medical Centre, Dr. Molewaterplein 40, 3015 GD Rotterdam, The Netherlands; 6 Centre for Cutaneous Research, Blizard Institute, Queen Mary University of London, Mile End Road, London E1 4NS, UK; 7 Department of Plastic and Reconstructive Surgery, Guangdong Second Provincial General Hospital, Guangzhou 510317, China; 8 Instituto Ivo Pitanguy, Hospital Santa Casa de Misericórdia Rio de Janeiro, Pontifícia Universidade Católica do Rio de Janeiro, Rio de Janeiro 20020-022, Brazil; giuseppesofo93@gmail.com; 9 Department of Oromaxillofacial Surgery, Ludwig-Maximilians University Munich, Lindwurmstraße 2A, 80337 Munich, Germany

**Keywords:** ChatGPT, artificial intelligence, medical state examination questions, indecisiveness, response consistency

## Abstract

(1) Background: As the field of artificial intelligence (AI) evolves, tools like ChatGPT are increasingly integrated into various domains of medicine, including medical education and research. Given the critical nature of medicine, it is of paramount importance that AI tools offer a high degree of reliability in the information they provide. (2) Methods: A total of *n* = 450 medical examination questions were manually entered into ChatGPT thrice, each for ChatGPT 3.5 and ChatGPT 4. The responses were collected, and their accuracy and consistency were statistically analyzed throughout the series of entries. (3) Results: ChatGPT 4 displayed a statistically significantly improved accuracy with 85.7% compared to that of 57.7% of ChatGPT 3.5 (*p* < 0.001). Furthermore, ChatGPT 4 was more consistent, correctly answering 77.8% across all rounds, a significant increase from the 44.9% observed from ChatGPT 3.5 (*p* < 0.001). (4) Conclusions: The findings underscore the increased accuracy and dependability of ChatGPT 4 in the context of medical education and potential clinical decision making. Nonetheless, the research emphasizes the indispensable nature of human-delivered healthcare and the vital role of continuous assessment in leveraging AI in medicine.

## 1. Introduction

In 1968, Marvin Minsky described “artificial intelligence” (AI) as “the science of making machines do things that would require intelligence if done by men” [1]. This definition, despite the passage of more than half a century, remains remarkably pertinent. The advent of AI technologies, especially in the form of advanced chatbots such as ChatGPT, has ushered in a new era of possibilities in various fields, including medicine. An extensive body of research underscores the significant value that ChatGPT brings to research and education, particularly within the medical domain. Its utility spans a broad spectrum of users, including physicians, healthcare workers, medical students, and even patients, assisting them in making informed, data-driven healthcare decisions [2,3,4,5,6].

ChatGPT represents just one facet of the burgeoning field of chatbots powered by Large Language Models (LLMs). Google’s Med-PaLM, for instance, marked a significant milestone by exceeding the benchmark score—over 60%—on questions modeled after the U.S. Medical Licensing Examination (USMLE), as detailed in a previous publication [7]. Following this achievement, Google introduced Med-PaLM 2, which has demonstrated considerable improvements over its predecessor, indicating significant performance advancements [8]. However, access to such advanced chatbots remains somewhat restricted, not being widely available to the general public.

Contrastingly, the widespread availability of LLMs like ChatGPT, Bard, and Bing has facilitated comprehensive comparative studies across various domains. For example, a study evaluating these models on case vignettes in Physiology highlighted ChatGPT 3.5’s superior performance over Bard and Bing, indicating its enhanced effectiveness in case-based learning [9]. Further, in a specialized comparison focusing on clinicopathological conferences regarding neurodegenerative disorders, both Google Bard and ChatGPT 3.5 were evaluated for their ability to deduce neuropathological diagnoses from clinical summaries. The findings revealed that both models accurately diagnosed 76% of cases, while ChatGPT 4 showed a higher accuracy rate, correctly identifying 84% of diagnoses [10]. Another study comparing ChatGPT 3.5, Google Bard, and Microsoft Bing in hematology-related cases noted distinct performance disparities, with ChatGPT achieving the highest accuracy [11].

The superior performance of ChatGPT, compared to other accessible alternatives, has been a crucial factor in our decision to conduct an in-depth investigation into this LLM’s effectiveness and capabilities. ChatGPT has demonstrated remarkable performances in various medical examinations, highlighting its potential as a significant educational and assessment tool in the medical field. A study by Oztermeli and Oztermeli in 2023 assessed ChatGPT’s performance in the five most recent medical specialty exams, revealing an average performance ranging from 54.3% to 70.9%, indicating proficient levels of understanding both clinical and basic science questions [12]. Similarly, Flores-Cohaila et al. reported that ChatGPT achieved an “expert-level performance” on the Peruvian National Licensing Medical Examination, with an accuracy of 86% with ChatGPT 4, followed by 77% with ChatGPT 3.5, significantly outperforming the examinee average of 55% [13]. This underscores ChatGPT’s potential to enhance medical education, especially in regions where access to educational resources may be limited.

However, acknowledging ChatGPT’s limitations is critical. Research has shown that earlier versions like ChatGPT 3.5 hovered at or near the passing threshold of approximately 60% for the USMLE. More specifically, Kung et al. demonstrated that ChatGPT 3.5 was unable to achieve a passing score for the Step 1 and Step 2CK examinations, scoring 55.8% and 59.1%, respectively, while it managed a passing score of 61.3% for the Step 3 examination [14]. Although the literature effectively highlights advancements in the accuracies of responses provided by ChatGPT in various medical education scenarios, the reliability of ChatGPT’s responses upon repeated queries remains uncertain. Ensuring high reliability in ChatGPT’s outputs is essential for users to trust its data-driven conclusions. Reliable information is of paramount importance in the medical field, where chatbots like ChatGPT can serve as a significant source of healthcare information for patients.

In this context, our aim was to assess the accuracy and reliability of ChatGPT in answering medical examination questions when queried multiple times. We also intended to contrast the performances of both commercially available versions, ChatGPT 3.5 and ChatGPT 4, to better understand their respective strengths and weaknesses.

The evolution of AI in medicine reflects a broader trend toward digital transformation in healthcare. AI-driven solutions, including chatbots, are increasingly being integrated into healthcare systems to support diagnostic processes, patient engagement, and personalized care plans. These technologies offer the promise of enhancing the efficiency and quality of healthcare services, reducing the burden on healthcare professionals, and facilitating patient access to reliable medical information.

Despite these promising developments, the integration of AI into healthcare raises ethical, legal, and social questions. Issues such as data privacy, algorithmic bias, and the need for transparent and explainable AI solutions are at the forefront of ongoing debates. As AI technologies become more embedded in healthcare, addressing these concerns is crucial to ensure that they serve the best interests of patients and healthcare providers alike.

In conclusion, the role of chatbots like ChatGPT in the medical field is an evolving narrative of technological innovation, offering both significant opportunities and challenges. As we continue to explore and understand the capabilities and limitations of these AI-driven tools, it is essential to approach their integration into healthcare with a balanced perspective, considering both their potential to transform medical education and patient care and the need to address the ethical, legal, and social implications of their use.

## 2. Materials and Methods

### 2.1. Question Bank Access and ChatGPT Data Entry

From 22 July to 18 August 2023, we accessed the Amboss© question bank and extracted 450 practice medical examination questions for the scope of this study. Prior to the initiation of the study, official permission for the use of the Amboss© question bank for research purposes was granted by Amboss© (Amboss GmbH, Berlin, Germany). The questions are in English and were specifically designed to aid medical students in their preparation for examination formats in the United States’ medical schools (e.g., shelf examinations, USMLE). Questions were selected from two categories within the question bank: “Shelf” and “Basic sciences”, each with 225 questions. Further, each category was divided into nine distinct subcategories, from which 25 questions were selected from each. The “Shelf” subcategories included Medicine, Surgery, Pediatrics, Obstetrics & Gynecology, Clinical Neurology, Psychiatry, Family Medicine, Emergency Medicine, and Ambulatory Care. The “Basic sciences” subcategories included Anatomy & Embryology, Behavioral Sciences, Biochemistry, Histology, Microbiology, Neurosciences, Pathology, Pharmacology, and Physiology. This categorization was strategically chosen to cover a broad spectrum of topics and specialties across clinical care (“Shelf”) and basic sciences (“Basic sciences”), ensuring comprehensive subject coverage of both more practical and theoretical aspects in medical education, respectively.

Questions were randomly selected using a number generator to guarantee an unbiased distribution, aiming for 25 questions per subcategory. The selected questions were then screened for their compatibility with ChatGPT (i.e., questions based only on text without any additional images) by three independent examiners (M.A.; C.C.H.; P.F.F.) and random checks were performed to ensure that none of the answers were indexed in major search engines. To assess the difficulty of the test questions, we utilized the proprietary rating system of the Amboss© question bank, which assigns a difficulty level based on the number of hammers (ranging from one to five). One hammer represented the easiest 20% of all questions, while five hammers indicated the most difficult 5% of questions [15].

**Figure 1:**
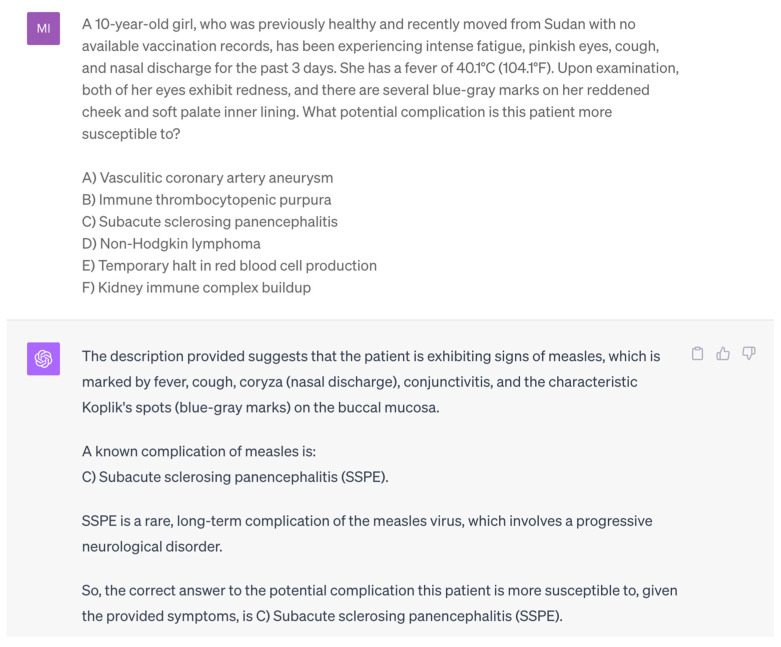
Manual entry of an exemplary question into ChatGPT.

**Figure 2:**
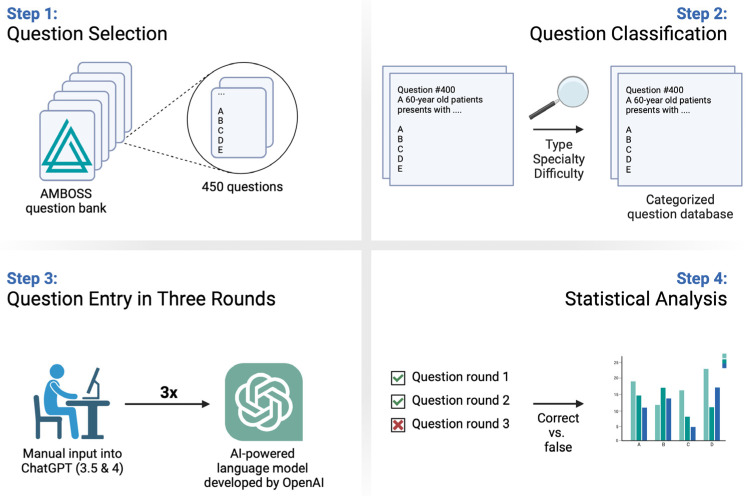
Comprehensive overview of the study’s workflow.

The questions were manually entered into ChatGPT by one examiner (P.F.F.), while strictly adhering to the original text, with a new chat session for each question to prevent memory bias [14]. Further, the authors refrained from using any additional prompts to ensure methodological standardization and minimize the risk of potential systematic errors by influencing the responses provided by ChatGPT through any preceding user prompts (Figure 1). Each question was entered thrice, each for ChatGPT 3.5 and ChatGPT 4. ChatGPT’s responses were recorded and entered into the corresponding Amboss© practice questions. Subsequently, data regarding the accuracy of the responses were meticulously gathered and collected in a separate excel spreadsheet. A comprehensive depiction of the entire study’s workflow can be found in Figure 2.

### 2.2. Statistical Analysis

Differences between the overall accuracies of ChatGPT 3.5 and ChatGPT 4 were calculated using a paired sample *t*-test while differences between question categories were calculated employing the independent student’s *t* test. Differences in the consistency between question entry rounds for each version were determined using Cochran’s Q test. The statistical analysis was conducted with IBM SPSS Statistics 27 (IBM, Armonk, NY, USA), and a two-tailed *p*-value of ≤0.05 was deemed to indicate statistical significance.

## 3. Results

### 3.1. General Test Question Characteristics and Overall Performance Statistics

A total of *n* = 2700 ChatGPT queries were manually entered with three queries for both ChatGPT 3.5 and ChatGPT 4 utilizing 450 practice questions (225 shelf and 225 basic science questions). The distribution of question difficulty was 22.0% with 1 hammer, 33.6% with 2 hammers, 28.0% with 3 hammers, 12.4% with 4 hammers, and 4.0% with 5 hammers. The overall accuracy for both versions was 71.7% (1936/2700 entries) while the accuracy was 57.7% (779/1350 entries) and 85.7% (1157/1350 entries) for ChatGPT 3.5 and ChatGPT 4, respectively (*p* < 0.001). When stratifying for question category, the overall performance for both versions was 71.9% (970/1350 entries) and 71.6% (966/1350 entries) for shelf and basic science questions, respectively (*p* = 0.918). Statistically significant differences in the accuracies of ChatGPT 3.5 and ChatGPT 4 were found for both shelf questions with 56.7% (766/1350 entries) and 87.0% (1174/1350 entries) with *p* < 0.001 and basic science questions with 58.7% (792/1350 entries) and 84.4% (1144/1350 entries) with *p* < 0.001. No statistically significant differences were found between both question categories for neither ChatGPT 3.5 with *p* = 0.635 nor for ChatGPT 4 with *p* = 0.374.

### 3.2. ChatGPT 3.5 Performance

In a detailed analysis of the response accuracy across three rounds of questions submitted to ChatGPT 3.5, the data revealed that the accuracy of correct responses was quantified at 57.6%, 57.1%, and 58.4% for the first, second, and third rounds, respectively. A statistical analysis indicated no significant variance among these percentages, with a *p*-value of 0.793, suggesting that the differences in accuracy rates between rounds were not statistically significant. Furthermore, the study found that in 44.9% of cases (202 out of 450 entries), ChatGPT consistently selected the correct answer across all three rounds of questions. In contrast, the correct answer was selected in two out of the three rounds in 11.6% of cases (52 out of 450 entries) and in only one round in 15.3% of cases (69 out of 450 entries). Notably, in 28.2% of cases (127 out of 450 entries), ChatGPT did not choose the correct answer in any of the rounds of entry. This comprehensive examination sheds light on the consistency and variability of ChatGPT 3.5’s performance in accurately responding to user queries across multiple rounds of interaction, highlighting areas for potential improvement in future iterations of the model.

### 3.3. ChatGPT 4 Performance

The accuracies of correct responses were measured to be 86.4%, 85.8%, and 84.9% for the first, second, and third round of questions entries into ChatGPT 4, respectively. No statistically significant difference between the three question entry rounds was found with *p* = 0.571. In 77.8% (350/450 entries) of cases, the correct answer was consistently chosen throughout the three rounds of entries while it was chosen in 9.1% (41/450 entries) in two rounds and in 5.6% (25/405 entries) in only one round. In 7.6% (34/450 entries) of cases, the correct answer was not chosen in any of the entry rounds. Response accuracies for both ChatGPT 3.5 and ChatGPT 4, when stratified by difficulty (i.e., number of Amboss© hammers), are summarized in Table 1.

## 4. Discussion

In the present study, we conducted an in-depth investigation to assess the accuracy and reliability of both commercially available versions ChatGPT 3.5 and ChatGPT 4 in answering medical examination questions sourced from the Amboss© question bank when queried multiple times. Our objective was to comprehensively evaluate ChatGPT 3.5’s and ChatGPT 4’s performances and their ability to respond to complex medical scenarios.

A major strength of our study was the utilization of an extensive practice question bank, kindly granted permission by Amboss©. This enabled us to categorize questions based on various medical categories, question types, and difficulties, facilitating an in-depth and insightful analysis of ChatGPT’s computational capabilities. These findings have important implications for the field of medical education and can contribute to further improvements in the training and development of AI systems in healthcare.

After the media hype clears, it becomes more and more apparent that ChatGPT is a boon and bane for state-of-the-art medical education. On the one hand, ChatGPT condenses medical knowledge, rendering the principles of clinical reasoning and patient examination a more compact body of knowledge. Subsequently, ChatGPT can help regulate the overflow of medical information and link the multitude of unconnected education resources, ultimately freeing up more time to master clinical topics. In contrast, ChatGPT poses the risk of developing a tunnel view of medical data and clinical knowledge by streamlining the information flow. Besides the complexity of its application, there have been concerns about the possibility of AI tools like ChatGPT being exploited to cheat or secure unfair benefits in medical exams. It is crucial to note that our research was focused on assessing ChatGPT’s efficacy as a study aid, rather than promoting its usage during actual exams. Despite these obstacles, the main message is the significance of incorporating ChatGPT into a broader educational strategy. This method should enhance AI-based education with conventional teaching methods, including textbooks, lectures, and personalized guidance from experts in the field. Such a blend not only offers a comprehensive learning experience, but also addresses potential issues of dependability and ethics that might arise from relying exclusively on AI tools for educational objectives.

Before ChatGPT was made available to the public, various research efforts were undertaken to assess how well AI models could respond to questions from medical licensing exams. For instance, Jin and colleagues observed that such models achieved a mere 37% accuracy when they tested them on a collection of 12,723 questions from Chinese medical licensing exams [16]. Similarly, Ha and their team found an even lower success rate of 29% after reviewing 454 questions from the 2019 USMLE Step 1 and Step 2 [17].

ChatGPT transcends the limitations of conventional question–answering approaches, marking a considerable advancement in the realm of online knowledge retrieval that benefits both medical professionals and the general public. Gilson and colleagues have shown that ChatGPT’s performance is on par with, or even exceeds, that of earlier models when dealing with questions of similar complexity and subject matter [18]. This underscores the model’s enhanced capability for producing precise answers through comprehensive analysis and medical insight.

The integration of AI tools like ChatGPT into the medical field has sparked a considerable amount of interest among healthcare professionals and medical educators. This interest stems from the potential of these tools to enhance the learning experience for medical students, aid physicians in keeping abreast of the latest medical knowledge, and offer patients access to information that can aid in understanding their health conditions. The performance of ChatGPT in responding to medical examination questions offers a window into its reliability and potential utility in real-world medical scenarios. Our research aligns with previous studies, presenting evidence that the accuracy and consistency of ChatGPT have seen significant improvements from version 3.5 to version 4, across various languages, medical specialties, and educational systems [13,19,20,21,22,23,24,25].

In our study, ChatGPT version 4 demonstrated a remarkable improvement in performance, with an 85.7% rate of correctly answered questions, compared to the 57.7% accuracy rate of its predecessor. This improvement is indicative not only of the model’s enhanced understanding of medical content, but also of its ability to provide consistent responses. Specifically, our study revealed that in 77.8% of cases, ChatGPT version 4 consistently chose the correct answer across all rounds of questioning, a substantial increase from the 44.9% consistency rate of ChatGPT 3.5.

The advancements in ChatGPT’s performance can be attributed to several key developments in its underlying technology. The model has benefited from the implementation of more sophisticated and efficient computing algorithms, enabling it to process and interpret larger datasets with greater precision. Moreover, the breadth and recency of the training data used have significantly enhanced the model’s comprehension capabilities. Notably, architectural changes have played a crucial role, with ChatGPT 4 rumored to possess a vastly larger number of parameters, potentially in the trillions, compared to the 175 billion parameters of its predecessor. This leap in architectural complexity, coupled with improvements in training methodologies, has led to noticeable enhancements in the model’s output. These include increased accuracy, a reduced tendency to generate implausible or irrelevant information, and a boost in the confidence levels of its responses, as our study effectively illustrates [26,27,28,29].

The implications of these advancements are profound, especially in the medical context, where reliability and accuracy are paramount. If the performance of ChatGPT on medical examination questions can serve as a surrogate for its reliability in providing medical information, it represents a significant step forward. However, despite these promising developments, it is essential for medical students, physicians, and patients to approach the information provided by AI tools like ChatGPT with caution. The inherent limitation of such tools is their reliance on the information provided to them, which means there is always a risk of omitting critical data necessary for making comprehensive, data-driven decisions. This underscores the importance of the continuous, rigorous evaluation of AI tools, particularly in critical domains like healthcare.

Nonetheless, the improvements in accuracy and consistency observed in ChatGPT 4’s performance are noteworthy. They highlight the tool’s potential to play a significant role in medical education and clinical practice, offering support in a variety of healthcare scenarios. Remarkably, ChatGPT 4’s test-taking capabilities have been shown to surpass those of many medical students, indicating that it meets the rigorous knowledge requirements essential for delivering high-quality healthcare. Furthermore, recent studies have suggested that AI can exhibit a degree of empathetic understanding, previously thought to be exclusive to human healthcare providers. This ability to engage with patients in a natural and effective manner suggests that AI can fulfill both the “hard skills” related to medical knowledge and the “soft skills” related to patient care [30,31,32].

However, it is crucial to recognize that the provision of healthcare extends beyond the fulfillment of quantifiable criteria. The nuanced judgment that comes from the personal assessments of patients, coupled with the empathetic connections developed through face-to-face interactions, remains irreplaceable. While AI technology offers valuable tools that can support various aspects of healthcare delivery, the unique contributions of human healthcare providers in offering compassionate, holistic care cannot be overlooked. As we continue to explore the integration of AI into medicine, it is vital to maintain a balanced perspective, embracing the potential of these technologies while acknowledging their limitations.

In conclusion, the enhanced performance of ChatGPT in responding to medical examination questions signals a promising advancement in the use of AI in healthcare. These improvements in accuracy and consistency reflect the model’s growing capability to support medical education and clinical decision making. However, the critical roles of human judgment and empathy in healthcare delivery underscore the importance of using AI as a complementary tool rather than a replacement for human healthcare providers. As AI technologies evolve, their potential to transform healthcare is undeniable, but their integration into the medical field must be approached with careful consideration of their strengths and limitations [33,34,35]. The future of healthcare will likely be characterized by a synergistic partnership between AI and human expertise, leveraging the best of both worlds to enhance patient care and medical education.

## 5. Limitations

This study offers insightful observations on the performance metrics of ChatGPT versions 3.5 and 4 in the context of medical examination queries, shedding light on their potential utility and accuracy in educational settings. However, it is essential to recognize several constraints that might impact the interpretation and applicability of the findings.

First and foremost, the study’s methodology might have been subject to selection bias due to the partially manual selection process utilized for gathering questions from the Amboss© question bank. Despite employing a random selection strategy, the deliberate omission of image-based questions could inadvertently influence the difficulty level and subject matter of the questions ultimately chosen for analysis. This bias could have skewed the study’s outcomes, making them less representative of the full spectrum of medical examination content. Moreover, the exclusive reliance on a single question bank and a specific question format could have limited the study’s relevance across the diverse landscape of medical education and clinical practice, potentially affecting the generalizability of the results to other examination types or educational tools, including key feature questions, essay formats, or objective-structured clinical examinations (OSCEs) [36,37].

Another notable limitation is the study’s focus on the accuracy and consistency of ChatGPT’s responses, while overlooking the AI’s reasoning processes or its capability to provide explanatory context for its answers. This omission is significant because the ability to understand the rationale behind a response is crucial in medical education and clinical decision making. By neglecting to examine the AI’s reasoning, this study may not have fully captured the educational value of ChatGPT, particularly in scenarios where comprehending the “why” behind an answer is as critical as the answer itself.

Furthermore, the research was conducted under controlled conditions, which may not accurately mirror the complex and unpredictable nature of real-life medical testing and patient care environments. The study’s standardized methodology, aimed at ensuring reproducibility, did not account for the potential impact of variable user interactions, question formulations, or the context of inquiries on the performance of ChatGPT. This limitation raises questions about the external validity of the findings, especially concerning the practical application of ChatGPT in genuine medical education and clinical settings.

Taking these limitations into account is crucial for a balanced interpretation of the study’s results. Future research endeavors should strive to mitigate these issues by broadening the scope of question types included in the analysis, diversifying the methods of question presentation, integrating an examination of the AI’s reasoning capabilities, and assessing the application of AI tools like ChatGPT in real-world clinical and educational contexts. Such comprehensive evaluations are vital for accurately determining the utility and reliability of AI in enhancing medical education and improving clinical decision-making processes. This approach will not only enrich our understanding of AI’s potential roles in healthcare, but also help in identifying areas where further development and refinement are needed to maximize the benefits of AI technologies in medical training and practice.

## 6. Conclusions

Our study aimed to rigorously evaluate the performances of ChatGPT versions 3.5 and 4 in the context of medical examination questions, focusing on their accuracy and consistency. The advent of AI in healthcare has opened new avenues for augmenting medical education and supporting clinical decision-making processes. With the healthcare sector increasingly relying on AI tools for various applications, the reliability and precision of these technologies are paramount. In our comparative analysis, ChatGPT 4 emerged as a significantly improved version, showcasing enhanced accuracy and consistency in its responses. This progression underscores the potential of advanced AI systems like ChatGPT 4 to serve as invaluable assets in the medical field, offering support in both educational environments and clinical settings.

The importance of high reliability in AI-generated responses cannot be overstated, particularly when these responses are utilized to assist physicians and patients in making informed healthcare decisions. Our findings suggest that as AI technologies like ChatGPT continue to evolve, they could play a pivotal role in shaping the future of medical consultation and patient care. Nevertheless, it is crucial to acknowledge that despite the significant benefits AI introduces to healthcare, it is designed to function as an augmentative tool rather than a standalone solution. The nuanced judgment, critical thinking, and interpersonal dynamics that human healthcare professionals bring to patient care are irreplaceable and remain at the core of effective healthcare delivery.

Therefore, while AI, exemplified by the advancements seen from ChatGPT 3.5 to ChatGPT 4, offers a multitude of opportunities for enhancing healthcare services, it is essential to maintain a balanced perspective. Emphasizing the augmentative nature of AI underscores the technology’s role in supporting, not supplanting, the expertise and empathetic care provided by human healthcare professionals. This balanced approach ensures that AI’s integration into healthcare augments the human elements that are fundamental to delivering compassionate and effective care, thereby maximizing the benefits of AI for patients and healthcare providers alike.

## Figures and Tables

**Table 1:** Summary of the accuracies of ChatGPT 3.5 and ChatGPT 4 through the three entry rounds, stratified by practice question difficulty.

		ChatGPT 3.5 First Round			ChatGPT 3.5 Second Round			ChatGPT 3.5 Third Round			ChatGPT 4 First Round			ChatGPT 4 Second Round			ChatGPT 4 Third Round		
		Wrong	Correct	Total	Wrong	Correct	Total	Wrong	Correct	Total	Wrong	Correct	Total	Wrong	Correct	Total	Wrong	Correct	Total
**Number of hammers (Difficulty)**	**Count**	17	82	99	20	79	99	14	85	99	4	95	99	2	97	99	6	93	99
	**Accuracy [%]**	17.2%	82.8%	100.0%	20.2%	79.8%	100.0%	14.10%	85.90%	100.00%	4.00%	96.00%	100.00%	2.00%	98.00%	100.00%	6.10%	93.90%	100.00%
	**Count**	62	89	151	64	87	151	62	89	151	7	144	151	8	143	151	12	139	151
	**Accuracy [%]**	41.1%	58.9%	100.0%	42.4%	57.6%	100.0%	41.10%	58.90%	100.00%	4.60%	95.40%	100.00%	5.30%	94.70%	100.00%	7.90%	92.10%	100.00%
	**Count**	63	63	126	60	66	126	63	63	126	21	105	126	27	99	126	21	105	126
	**Accuracy [%]**	50.0%	50.0%	100.0%	47.6%	52.4%	100.0%	50.00%	50.00%	100.00%	16.70%	83.30%	100.00%	21.40%	78.60%	100.00%	16.70%	83.30%	100.00%
	**Count**	36	20	56	36	20	56	34	22	56	20	36	56	17	39	56	20	36	56
	**Accuracy [%]**	64.3%	35.7%	100.0%	64.3%	35.7%	100.0%	60.70%	39.30%	100.00%	35.70%	64.30%	100.00%	30.40%	69.60%	100.00%	35.70%	64.30%	100.00%
	**Count**	13	5	18	13	5	18	14	4	18	9	9	18	10	8	18	9	9	18
	**Accuracy [%]**	72.2%	27.8%	100.0%	72.2%	27.8%	100.0%	77.80%	22.20%	100.00%	50.00%	50.00%	100.00%	55.60%	44.40%	100.00%	50.00%	50.00%	100.00%
**Total**	**Count**	191	259	450	193	257	450	187	263	450	61	389	450	64	386	450	68	382	450
	**Accuracy [%]**	42.4%	57.6%	100.0%	42.9%	57.1%	100.0%	41.60%	58.40%	100.00%	13.60%	86.40%	100.00%	14.20%	85.80%	100.00%	15.10%	84.90%	100.00%

## Data Availability

The data presented in this study are available upon request from the corresponding author.

## References

[1] Stonier T., Stonier T. (1992). The Evolution of Machine Intelligence. Beyond Information: The Natural History of Intelligence.

[2] Hoch C.C., Wollenberg B., Lüers J.-C., Knoedler S., Knoedler L., Frank K., Cotofana S., Alfertshofer M. (2023). ChatGPT’s quiz skills in different otolaryngology subspecialties: An analysis of 2576 single-choice and multiple-choice board certification preparation questions. Eur. Arch. Oto-Rhino-Laryngol..

[3] Alfertshofer M., Hoch C.C., Funk P.F., Hollmann K., Wollenberg B., Knoedler S., Knoedler L. (2023). Sailing the Seven Seas: A Multinational Comparison of ChatGPT’s Performance on Medical Licensing Examinations. Ann. Biomed. Eng..

[4] Dave T., Athaluri S.A., Singh S. (2023). ChatGPT in Medicine: An Overview of Its Applications, Advantages, Limitations, Future Prospects, and Ethical Considerations. Front. Artif. Intell..

[5] Tangadulrat P., Sono S., Tangtrakulwanich B. (2023). Using ChatGPT for Clinical Practice and Medical Education: Cross-Sectional Survey of Medical Students’ and Physicians’ Perceptions. JMIR Med. Educ..

[6] Ahmed Y. (2023). Utilization of ChatGPT in Medical Education: Applications and Implications for Curriculum Enhancement. Acta Inform. Medica.

[7] Singhal K., Azizi S., Tu T., Mahdavi S.S., Wei J., Chung H.W., Scales N., Tanwani A., Cole-Lewis H., Pfohl S. (2023). Large language models encode clinical knowledge. Nature.

[8] Singhal K., Tu T., Gottweis J., Sayres R., Wulczyn E., Hou L., Clark K., Pfohl S., Cole-Lewis H., Neal D. (2023). Towards Expert-Level Medical Question Answering with Large Language Models. arXiv.

[9] Dhanvijay A.K.D., Pinjar M.J., Dhokane N., Sorte S.R., Kumari A., Mondal H. (2023). Performance of Large Language Models (ChatGPT, Bing Search, and Google Bard) in Solving Case Vignettes in Physiology. Cureus.

[10] Koga S., Martin N.B., Dickson D.W. (2023). Evaluating the performance of large language models: ChatGPT and Google Bard in generating differential diagnoses in clinicopathological conferences of neurodegenerative disorders. Brain Pathol..

[11] Kumari A., Singh A., Singh S.K., Juhi A., Dhanvijay A.K.D., Pinjar M.J., Mondal H., Dhanvijay A.K. (2023). Large Language Models in Hematology Case Solving: A Comparative Study of ChatGPT-3.5, Google Bard, and Microsoft Bing. Cureus.

[12] Oztermeli A.D., Oztermeli A. (2023). ChatGPT Performance in the Medical Specialty Exam: An Observational Study. Medicine.

[13] Flores-Cohaila J.A., García-Vicente A., Vizcarra-Jiménez S.F., De la Cruz-Galán J.P., Gutiérrez-Arratia J.D., Torres B.G.Q., Taype-Rondan A. (2023). Performance of ChatGPT on the Peruvian National Licensing Medical Examination: Cross-Sectional Study. JMIR Med. Educ..

[14] Kung T.H., Cheatham M., Medenilla A., Sillos C., De Leon L., Elepaño C., Madriaga M., Aggabao R., Diaz-Candido G., Maningo J. (2023). Performance of ChatGPT on USMLE: Potential for AI-assisted medical education using large language models. PLoS Digit. Health.

[15] Amboss©. Question Difficulty. https://support.amboss.com/hc/en-us/articles/360035679652-Question-difficulty.

[16] Jin D., Pan E., Oufattole N., Weng W.-H., Fang H., Szolovits P. (2021). What disease does this patient have? A large-scale open domain question answering dataset from medical exams. Appl. Sci..

[17] Ha L.A., Yaneva V. (2019). Automatic Question Answering for Medical MCQs: Can It Go Further Than Information Retrieval?. Proceedings of the International Conference on Recent Advances in Natural Language Processing (RANLP 2019).

[18] Gilson A., Safranek C.W., Huang T., Socrates V., Chi L., Taylor R.A., Chartash D. (2023). How Does ChatGPT Perform on the United States Medical Licensing Examination? The Implications of Large Language Models for Medical Education and Knowledge Assessment. JMIR Med. Educ..

[19] Frosolini A., Franz L., Benedetti S., Vaira L.A., de Filippis C., Gennaro P., Marioni G., Gabriele G. (2023). Assessing the accuracy of ChatGPT references in head and neck and ENT disciplines. Eur. Arch. Oto-Rhino-Laryngol..

[20] Knoedler L., Alfertshofer M., Knoedler S., Hoch C.C., Funk P.F., Cotofana S., Maheta B., Frank K., Brébant V., Prantl L. (2024). Pure Wisdom or Potemkin Villages? A Comparison of ChatGPT 3.5 and ChatGPT 4 on USMLE Step 3 Style Questions: Quantitative Analysis. JMIR Med. Educ..

[21] Massey P.A., Montgomery C., Zhang A.S. (2023). Comparison of ChatGPT–3.5, ChatGPT-4, and Orthopaedic Resident Performance on Orthopaedic Assessment Examinations. JAAOS-J. Am. Acad. Orthop. Surg..

[22] Brin D., Sorin V., Vaid A., Soroush A., Glicksberg B.S., Charney A.W., Nadkarni G., Klang E. (2023). Comparing ChatGPT and GPT-4 performance in USMLE soft skill assessments. Sci. Rep..

[23] Moshirfar M., Altaf A.W., Stoakes I.M., Tuttle J.J., Hoopes P.C. (2023). Artificial Intelligence in Ophthalmology: A Comparative Analysis of GPT-3.5, GPT-4, and Human Expertise in Answering StatPearls Questions. Cureus.

[24] Takagi S., Watari T., Erabi A., Sakaguchi K. (2023). Performance of GPT-3.5 and GPT-4 on the Japanese Medical Licensing Examination: Comparison Study. JMIR Med. Educ..

[25] Strong E., DiGiammarino A., Weng Y., Kumar A., Hosamani P., Hom J., Chen J.H. (2023). Chatbot vs Medical Student Performance on Free-Response Clinical Reasoning Examinations. JAMA Intern. Med..

[26] Martindale J. (2023). GPT-4 vs. GPT-3.5: How much Difference is There? Digital Trends. https://www.digitaltrends.com/computing/gpt-4-vs-gpt-35/.

[27] Bastian M. (2023). GPT-4 has More Than a Trillion Parameters—Report. The Decoder. https://the-decoder.com/gpt-4-has-a-trillion-parameters/.

[28] Zaitsu W., Jin M. (2023). Distinguishing ChatGPT(-3.5, -4)-generated and human-written papers through Japanese stylometric analysis. PLoS ONE.

[29] Rudolph J., Tan S., Tan S. (2023). ChatGPT: Bullshit spewer or the end of traditional assessments in higher education?. J. Appl. Learn. Teach..

[30] Norcini J., Grabovsky I., Barone M.A., Anderson M.B., Pandian R.S., Mechaber A.J. (2024). The Associations Between United States Medical Licensing Examination Performance and Outcomes of Patient Care. Acad. Med..

[31] Howe P.D.L., Fay N., Saletta M., Hovy E. (2023). ChatGPT’s advice is perceived as better than that of professional advice columnists. Front. Psychol..

[32] Elyoseph Z., Hadar-Shoval D., Asraf K., Lvovsky M. (2023). ChatGPT outperforms humans in emotional awareness evaluations. Front. Psychol..

[33] Powell J. (2019). Trust Me, I’m a Chatbot: How Artificial Intelligence in Health Care Fails the Turing Test. J. Med. Internet Res..

[34] Yokoi R., Eguchi Y., Fujita T., Nakayachi K. (2021). Artificial Intelligence Is Trusted Less than a Doctor in Medical Treatment Decisions: Influence of Perceived Care and Value Similarity. Int. J. Hum. Comput. Interact..

[35] Lee J. (2020). Is Artificial Intelligence Better Than Human Clinicians in Predicting Patient Outcomes?. J. Med. Internet. Res..

[36] Al-Wardy N.M. (2010). Assessment Methods in Undergraduate Medical Education. Sultan Qaboos Univ. Med. J..

[37] Tabish S.A. (2008). Assessment Methods in Medical Education. Int. J. Health Sci..

